# Incidence rates of surgically treated idiopathic carpal tunnel syndrome in blue- and white-collar workers and housewives in Tuscany, Italy

**DOI:** 10.1136/oem.2008.040212

**Published:** 2009-03-01

**Authors:** S Mattioli, A Baldasseroni, S Curti, R M T Cooke, A Mandes, F Zanardi, A Farioli, E Buiatti, G Campo, F S Violante

**Affiliations:** 1 Occupational Medicine Unit, Dipartimento di Medicina Interna, dell’Invecchiamento e Malattie Nefrologiche, University of Bologna, Bologna, Italy; 2 Tuscany Regional Centre for Occupational Injuries and Diseases (CeRIMP), Florence, Italy; 3 Tuscany Regional Health Care Agency, Florence, Italy; 4 Dipartimento Processi Organizzativi, National Institute of Occupational Safety and Prevention (ISPESL), Rome, Italy

## Abstract

**Objectives:**

Rates of surgically treated carpal tunnel syndrome (CTS) among blue- and white-collar workers and housewives in the general population were compared.

**Methods:**

Surgically treated cases of idiopathic CTS were investigated among 25–59-year-old residents of Tuscany, Italy, during 1997–2000, based on obligatory discharge records from all Italian public/private hospitals, archived according to residence on Tuscany’s regional database. Population data were extracted from the 2001 census.

**Results:**

After excluding repeat admissions, 8801 eligible cases were identified. Age-standardised rates (per 100 000 person-years) of surgical CTS were: “blue-collar women”, 367.8; “white-collar women”, 88.1; “housewives”, 334.5; “blue-collar men”, 73.5; and “white-collar men”, 15.3. Compared with reference categories (same-sex white-collar workers): female blue-collar workers experienced a 4.2-fold higher standardised rate; housewives, a 3.8-fold excess; and male blue-collar workers, a 4.8-fold excess (all p<0.001). Male and female blue-collar workers showed approximately three to sevenfold higher age-specific rates compared to their white-collar counterparts (all p<0.001). Housewives’ rates were similar to those of blue-collar female workers up to 40–44 years of age, after which they were significantly lower (p<0.002). At all ages, housewives’ rates were much higher (p<0.001) than those of white-collar women.

**Conclusions:**

Surgically treated CTS was three to seven times more common (depending on age/gender) in blue-collar than in white-collar workers, which is difficult to explain by differences in body weight or other individual factors. Thus, occupational risk factors seem relevant throughout working life. The high rates for full-time housewives suggest that domestic chores should be investigated as a possible risk factor for CTS.

Carpal tunnel syndrome (CTS) is a disabling condition^1^ ^2^ which accounts for many years of lost productivity.^3^ Biomechanical overload associated with repetitive, forceful manual work is a major risk factor for CTS.^4^ CTS affects women more frequently than men, with peak incidence occurring during the perimenopause (in contrast to a more gradual age-related increase in men).^5^ Being overweight and biomechanical overload accompanying repetitive forceful manual tasks are believed to be relevant risk factors for CTS.^6^

A survey of the general population in southern Sweden found that blue-collar workers had approximately double the risk of symptomatic CTS (and also of electrophysiologically confirmed CTS) compared to white-collar workers.^7^ Cross-sectional studies have suggested that manual workers in a variety of occupations are at increased risk of CTS, including those working in slaughterhouses, on poultry farms, on assembly lines, in the clothing industry, in supermarkets, packing food and cutting/drilling stone, as well as certain white-collar workers whose jobs involve biomechanical stresses to the hand/wrist (dental hygienists and those using a computer mouse intensively^8^). A case–control study of job classes additionally suggested raised risks for cooks.^9^ A study of rates of first surgery for CTS in the general population of Montreal, Canada using the provincial health insurance database^10^ was able to identify seven at-risk job categories: housekeepers/cleaners, data-processing operators, material handlers, food/beverage processing workers, service workers, male lorry/bus drivers and (more generally) all manual workers. However, the entity of age-related risks associated with biomechanical exposures encountered during various types of manual work are debated.^1^ ^8^ ^11^ ^12^ Furthermore, the possible relevance of domestic chores^13^ ^14^ has been little studied.

Surgery is the treatment of choice for cases of severe chronic CTS refractory to conservative approaches.^15^ We used the administrative records of hospital treated patients from Tuscany, Italy to assess the incidence rates of hospital treated idiopathic CTS among blue- and white-collar workers and full-time housewives.

## METHODS

### Setting and study design

Using hospital discharge records and census data, we evaluated age- and sex-specific incidence rates of surgically treated CTS among blue- and white-collar workers and full-time housewives in the general population of Tuscany (3.5 million inhabitants), Italy in 1997–2000. In Italy during this period, diagnosis of CTS supported by median nerve conduction studies was considered a prerequisite for surgical treatment, and out-of-hospital CTS surgery was almost completely absent due to reimbursement regulations. In Italy, all public/private hospitals are obliged to provide individual, codified discharge records, even after day treatment. These discharge records are stored in databases of the patients’ region of residence, irrespective of hospital location. The discharge records of hospitals within the administrative Region of Tuscany (Regione Toscana) from the period also contain codified information on the generic occupational categories listed in table 1, including a specific code for full-time housewives. This information allowed us to classify patients as white-collar workers, blue-collar workers (including borderline “mixed-collar” workers), full-time housewives, and others. Accordingly, we reviewed the records of all patients resident in the Region of Tuscany with a discharge record issued by any Italian hospital between 1997 and 2000 bearing a principal diagnosis of CTS (ICD-9 code 354.0) coupled with specific surgical treatment (Diagnosis Related Group code 006, “Carpal Tunnel Release”). Repeated outpatient/inpatient admissions with a principal diagnosis of CTS during the study period were excluded.

**Table 1:** Employment categories of actively working patients with surgically treated idiopathic carpal tunnel syndrome (aged 25–59 years)

	Women (n = 7535)	Men (n = 1266)	Overall (n = 8801)
White-collar workers	886	189	1075
Managers	10	4	14
Self-employed professionals	49	26	75
Entrepreneurs	22	12	34
Clerical workers	570	102	672
Associate professionals	235	45	280
Blue-collar (and “mixed-collar”)	3330	1077	4407
workers			
Skilled/unskilled manual workers*	1011	413	1424
Service workers†	1498	407	1905
Home-based workers†	154	1	155
Self-employed workers†	667	256	923
Housewives	3319	–	3319

*Includes apprentices; †categories may include some “mixed-collar” workers.

**Figure 1:**
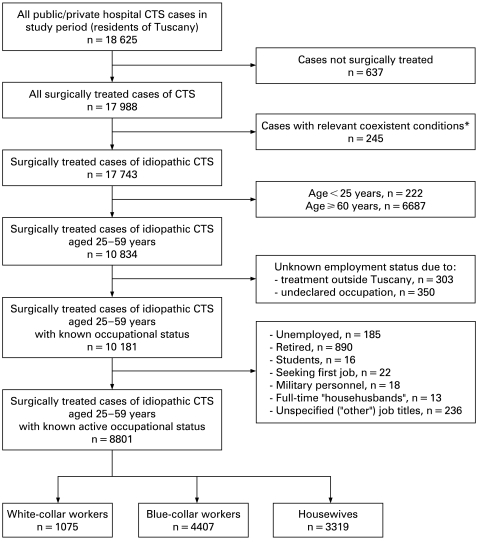
Flow chart of cases meeting the study eligibility criteria. *Cases also bearing the following ICD-9 codes were considered not to be idiopathic: 245 (hypothyroidism, thyroiditis), 250 (diabetes mellitus), 274.0 (gout), 277.3 (amyloidosis), 278 (overweight/obesity), 646.8 and 646.9 (complications of pregnancy), 710 (connective tissue diseases), 714 (rheumatoid arthritis), 715.3 and 715.4 (osteoarthritis of the hand/forearm), 813.4 (wrist fractures), 955 (shoulder/upper limb peripheral nerve injuries) and V22 (pregnancy). CTS, carpal tunnel syndrome.

To restrict the study to idiopathic CTS, we excluded cases with secondary (ie, coexisting) diagnoses of conditions thought to be associated with a increased risk of CTS,^16^^–^18 namely hypothyroidism (ICD-9 codes 243, 244), thyroiditis (245), diabetes mellitus (250), gout (274.0), amyloidosis (277.3), overweight/obesity (278), complications of pregnancy (646.8, 646.9), connective tissue diseases (710), rheumatoid arthritis (714), osteoarthritis of the hand/forearm (715.3, 715.4), wrist fractures (813.4), shoulder/upper limb peripheral nerve injuries (955) and pregnancy (V22). Because of limited numbers of cases in the youngest age groups and selection bias considerations related to “retired” occupational status, we decided to restrict the study to subjects aged 25–59 years. As shown in fig 1, we also excluded members of the armed forces (due to white-/blue-collar classification difficulties), students, full-time “househusbands”, cases with undeclared/unknown employment status (due to treatment outside Tuscany, etc), unemployed or retired subjects (due to lack of information about previous occupational status), first job seekers, and those with “other” (unspecified) job titles. We grouped the job titles reported on the hospital discharge records into occupational categories and classified them into blue-collar (including “mixed-collar”), white-collar and housewives, as shown in table 1.

Population data for the age groups of interest in the study area were extracted from the most recent national census, which was conducted in 2001; of note, we are not aware of any major workforce changes in Tuscany between 1997 and 2001. To facilitate white-/blue-collar classification we chose to use census data that had been tabulated based on the ISTAT-2001 Classification of Occupations.^19^ We classified the following groupings as white-collar: clerks, technicians and associate professionals, highly skilled professionals, directors and managers. The following groupings were classified as blue-collar (including “mixed-collar”): unskilled workers, production-line/machine workers and drivers, skilled manual workers, farm/horticulture workers, service workers and retailers. The only other listed employment grouping was members of the armed forces, a category which was excluded from the study. Numbers of full-time housewives in the general population were extracted from ISTAT’s “non-workforce” classification, which includes a specific “housewife” category.^19^

### Statistical analysis

We calculated age-sex specific incidence rates (per 100 000 person-years) and standardised rates (age adjusted by the Standard European Population proposed by WHO)^20^ with respect to occupational status. Age-sex specific rate ratios for blue-collar workers and housewives were calculated taking white-collar workers as the reference category. For both rates and rate ratios, we calculated 95% confidence intervals (95% CI) to take into account the sampling error related to a restricted (4-year) time period. For two-way comparisons, we used the z test to test the null hypothesis that rates in groups of interest were equal.^21^ To test age-related trends in incidence rates, we used the score test and reported rate ratio estimates for each one unit increase in age class^22^; for the rate ratios, we used a non-parametric test for trend across ordered groups.^23^ Since the hospital discharge records database did not permit identification of patients in years before the observation period, we performed a sensitivity analysis by excluding the first 2 years of the observation period (ie, 1997 and 1998) to explore the possibility that the main analysis might have been distorted by the inclusion of some prevalent cases. Stata 9.0 SE (Stata, College Station, TX) was used for analysis with a significance level of 0.05.

## RESULTS

The process used to identify surgically treated cases of CTS meeting the study eligibility criteria is described in fig 1. Data regarding employment status were available for 10 181 (94%) of the 10 834 surgically treated patients aged 25–59 years with idiopathic CTS. A total of 8801 cases with known active occupational status that satisfied the study eligibility criteria entered the main analysis. Table 1 shows the distribution of white-, blue- and mixed-collar job categories and housewives among the cases.

**Figure 2:**
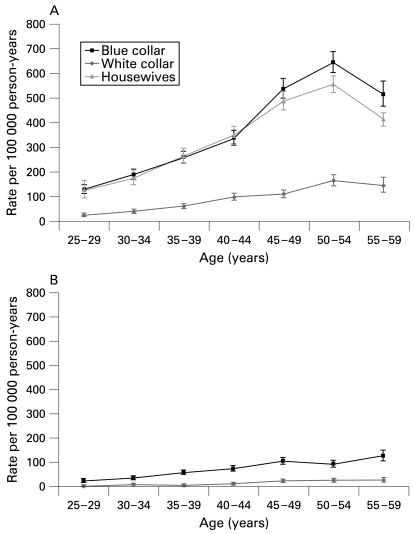
Age-specific incidence rates of surgically treated idiopathic carpal tunnel syndrome according to occupational category in women (A) and men (B).

Overall age-standardised incidence rates of surgically treated CTS (per 100 000 person-years) were 255.4 (95% CI 249.6 to 261.2) for women and 46.4 (95% CI 43.8 to 49.0) for men. Among women, age-standardised rates were 367.8 (95% CI 355.1 to 380.5) for blue-collar workers, 334.4 (95% CI 322.0 to 346.9) for housewives, and 88.1 (95% CI 81.9 to 94.2) for white-collar workers. Thus, with respect to their white-collar counterparts, female blue-collar workers had a 4.2-fold higher rate of surgically treated CTS, and housewives had a 3.8-fold excess. Among men, the age-standardised rates were 73.5 (95% CI 68.9 to 78.0) for blue-collar workers and 15.3 (95% CI 13.1 to 17.5) for white-collar workers. Thus, male blue-collar workers experienced a 4.8-fold higher rate of surgically treated CTS with respect to their white-collar counterparts. Table 2 and fig 2 report age-specific rates for women and men according to occupational categories. Of note, sensitivity analysis based on the last 2 years of the observation period generated curves that were very similar to those of the main analysis (data not shown), suggesting that distortion due to inclusion of some prevalent cases was unlikely. All the curves shown in fig 2 broadly displayed expected age-related patterns of CTS incidence,^24^ characterised by a peak around the ages of 50–54 years in women, and a more progressive rise in men. Highly significant age-related trends in incidence rates were apparent in all the occupational categories under study: rate ratios for each 5-year age class unit were 1.30 (95% CI 1.27 to 1.32) for female blue-collar workers, 1.35 (95% CI 1.30 to 1.40) for female white-collar workers, 1.16 (95% CI 1.14 to 1.18) for housewives, 1.27 (95% CI 1.23 to 1.31) for male blue-collar workers, and 1.29 (95% CI 1.19 to 1.39) for male white-collar workers (always p<0.001 in the score test for trend). Both female and male blue-collar workers showed higher age-specific rates with respect to white-collar workers at all ages (always p<0.001). No difference was apparent between blue-collar female workers and housewives’ rates up to the age of 45–49 years, after which blue-collar female workers’ rates were significantly higher (p<0.002 in both age classes).

**Table 2:** Age-sex-specific rates per 100 000 person-years (with 95% CI) of surgically treated idiopathic carpal tunnel syndrome according to occupational category, together with absolute numbers [cases/at-risk subjects]

Age (years)	Women			Men	
	Blue-collar workers	White-collar workers	Full-time housewives	Blue-collar workers	White-collar workers
25–29	127.4 (110.5 to 146.9)	24.6 (18.0 to 33.7)	124.5 (94.6 to 163.9)	26.2 (20.6 to 33.4)	4.5 (2.0 to 9.9)
	[190/149 132]	[39/158 240]	[51/40 952]	[65/247 904]	[6/134 288]
30–34	187.2 (167.8 to 208.9)	38.1 (30.4 to 47.8)	173.8 (146.8 to 205.7)	37.8 (31.5 to 45.5)	10.7 (6.8 to 16.7)
	[320/170 952]	[75/196 876]	[135/77 688]	[113/298 668]	[19/178 036]
35–39	257.5 (234.0 to 283.4)	58.4 (49.0 to 69.7)	261.3 (232.6 to 293.5)	59.3 (51.0 to 68.9)	8.3 (5.2 to 13.2)
	[419/162 728]	[124/212 272]	[284/108 708]	[170/286 732]	[18/216 388]
40–44	335.0 (306.4 to 366.3)	95.6 (82.6 to 110.8)	345.6 (312.9 to 381.7)	75.2 (65.1 to 86.8)	12.4 (8.4 to 18.4)
	[483/144 164]	[178/186 136]	[389/112 560]	[185/246 052]	[25/200 976]
45–49	534.8 (495.6 to 577.0)	106.2 (91.7 to 122.9)	485.3 (448.6 to 525.1)	104.7 (91.7 to 119.6)	22.4 (16.7 to 30.0)
	[665/124 348]	[179/168 588]	[620/127 744]	[218/208 228]	[45/201 196]
50–54	641.8 (600.1 to 686.4)	160.8 (140.1 to 184.5)	552.1 (518.9 to 587.3)	92.6 (80.5 to 106.5)	25.6 (19.2 to 34.0)
	[851/132 596]	[203/126 260]	[1003/181 680]	[196/211 760]	[47/183 872]
55–59	511.3 (463.7 to 563.8)	140.1 (113.7 to 172.7)	409.2 (382.4 to 437.9)	125.9 (106.0 to 149.5)	24.8 (17.2 to 35.6)
	[402/78 624]	[88/62 804]	[837/204 524]	[130/103 244]	[29/117 112]

**Figure 3:**
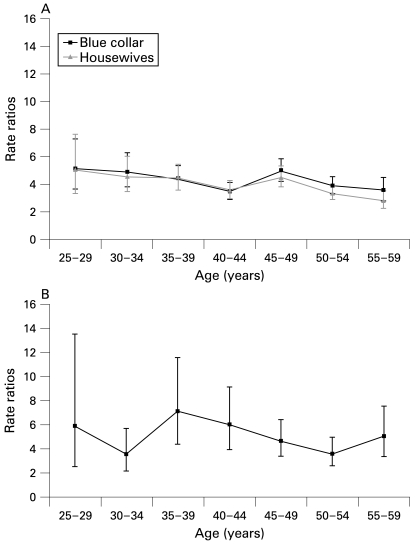
Age-sex specific rate ratios (A, women; B, men) for blue-collar workers and full-time housewives with respect to white-collar workers.

Table 3 and fig 3 report age-sex specific rate ratios of blue-collar workers and (for women only) housewives with respect to white-collar workers. The shapes of the age-related rate ratio curves were again remarkably similar for female blue-collar workers and housewives, apparently characterised by an overall decline, except for a transient peak at 45–49 years (tests for trend: blue-collar workers, p = 0.14; housewives, p = 0.04). By contrast, the rate ratio curve for male blue-collar workers showed no sign of a statistical trend (p = 0.66).

**Table 3:** Age-sex specific rate ratios (with 95% CI) for blue-collar workers and full-time housewives (with respect to white-collar workers)

Age(years)	Women		Men
	Blue-collar workers	Housewives	Blue-collar workers
25–29	5.2 (3.7 to 7.3)	5.1 (3.3 to 7.7)	5.9 (2.5 to 13.5)
30–34	4.9 (3.8 to 6.3)	4.6 (3.4 to 6.0)	3.5 (2.2 to 5.8)
35–39	4.4 (3.6 to 5.4)	4.5 (3.6 to 5.5)	7.1 (4.4 to 11.6)
40–44	3.5 (3.0 to 4.2)	3.6 (3.0 to 4.3)	6.0 (4.0 to 9.2)
45–49	5.0 (4.3 to 5.9)	4.6 (3.9 to 5.4)	4.7 (3.4 to 6.5)
50–54	4.0 (3.4 to 4.7)	3.4 (3.0 to 4.0)	3.6 (2.6 to 5.0)
55–59	3.6 (2.9 to 4.6)	2.9 (2.3 to 3.6)	5.1 (3.4 to 7.6)

All statistical comparisons with white-collar counterparts (reference categories) were highly significant (p<0.001, z test).

## DISCUSSION

This study indicates that surgically treated idiopathic CTS may be as much as three to seven times more common (depending on age and gender) in blue-collar workers than in their white-collar counterparts. It must be stressed that we were unable to adjust for factors other than age and sex. Nevertheless, we believe that such high excess risks among male and female blue-collar workers of various ages would be difficult to attribute to only moderate differences^25^ in body weight and height, or other individual factors such as familiality. Moreover, housewives showed remarkably similar age-related patterns to female blue-collar workers, with rate ratios several times higher than those of female white-collar workers.

Unsurprisingly,^26^ ^27^ perimenopausal incidence peaks were recorded (at 50–54 years of age) for women in all three occupational categories. The findings regarding relative risk of surgically treated CTS in blue-collar versus white-collar workers are broadly in line with the results of a study on the incidence of surgically treated CTS in the general population of Montreal, Canada, which used a similar case definition,^10^ as well as with data available from two different studies from southern Sweden (a population-based survey of clinically/electrophysiologically diagnosed CTS^28^ and a randomised trial of surgical treatment strategies^7^). Nevertheless, female white-collar workers tended to have incidence rates that were either approximately equivalent to or higher than those of male blue-collar workers (table 2, fig 2). This observation underlines the importance of female gender and hormonal factors as predisposing risk factors for CTS.^11^ ^29^

The primary explanation for the greatly increased risk of surgically treated CTS recorded among male and female blue-collar workers at all ages is likely to be exposure to manual work which involves taxing hand-wrist activities, such as prolonged, highly repetitive wrist flexion/extension, forceful grip in awkward postures, and use of hand-held vibratory tools.^1^ ^8^ ^30^ These biomechanical exposures are encountered in many blue-collar settings and are thought to be relevant occupational risk factors for CTS. Contributing factors could include the somewhat higher average body weight among individuals of lower socioeconomic status,^31^ since overweight is thought to be a further risk factor for CTS.^25^ However, based on estimates of the (more limited) entity of the risk associated with overweight,^6^ it seems likely that body weight would only explain a small fraction of the discrepancy between blue-collar and white-collar workers.

It might be argued that blue-collar workers are more likely to access surgical treatment due to their greater need to conserve manual fitness. However, in a comparison of surgically treated and electromyographically diagnosed CTS patients in the Tuscan town of Siena,^32^ the socioeconomic characteristics of the two groups appeared to be broadly similar, and the surgically treated patients actually seemed to have a slightly higher level of education. It also seems unlikely that malingering could have been a major contributing factor as there are many ways of gaining days off work without recourse to surgical treatment, and any “invented” cases would have to get through a preoperative clinical filter.

The characteristics of the Tuscan coding system also allowed us to evaluate the risks associated with unemployed housewife status. The remarkably similar patterns of surgically treated CTS among Tuscan housewives and female blue-collar workers highlights the possibly aetiological relevance of domestic work. In a case–control study of determinants of surgically treated CTS, we found that full-time housewives again appeared to have an approximately fourfold excess risk in comparison with white-collar workers (unpublished data). Furthermore, in an evaluation of associations between marital status and surgically treated CTS in several regions of Italy,^33^ we noted higher disease rates among married women. These observations encourage us to re-evaluate the hypothesis that domestic work may be a relevant risk factor. The results of a Chinese case–control study indicated that household tasks of Beijing women were associated with a raised risk of clinically/instrumentally diagnosed CTS.^14^ A survey of the biomechanical loads encountered in housewives’ routine domestic tasks revealed repetitive movements, frequently accompanied by high levels of hand/arm force and awkward postures.^34^ Remarkably, in the population-based study of the incidence of surgically treated CTS in Montreal,^10^ two of the seven specific at-risk occupations identified were housekeepers/cleaners and child care workers (both involving tasks commonly undertaken by housewives). Similar age-related trends were found for housewives and female blue-collar workers in terms of both the incidence of surgically treated CTS (fig 2) and rate ratios with respect to white-collar workers (fig 3). The age-related incidence curve for full-time housewives never dropped much below that of female blue-collar workers, and remained much higher than that of the female white-collar workers; these observations were reflected in the rate ratio curves of housewives and female blue-collar workers, which were almost parallel. Taken together, these findings suggest it is worth studying domestic work as a possible risk factor for CTS.^33^

### Study strengths and limitations

The study design allowed estimation of age-related risks associated with blue-collar employment and housewife status in the general population. We are confident that use of the hospital discharge records which every Italian public/private hospital is obliged to supply to local administrations allowed us to identify the vast majority of surgically treated cases of idiopathic CTS which occurred among residents of Tuscany between 1997 and 2000, when out-of-hospital CTS surgery was almost completely absent in Italy and going abroad for treatment was presumably not a widely considered option for CTS. Nevertheless, subsequent exclusion of retired subjects from the main analysis due to lack of information on occupational history limits the validity of the results around retirement age. Furthermore, our attempt to restrict the numerators to cases of “idiopathic” CTS may have been affected by under-reporting of concomitant conditions in the discharge records, perhaps especially in the context of patients with lower educational status. This factor might have led to slight overestimates of (i) the overall rates of surgically treated idiopathic CTS, and (ii) the rate ratios for blue-collar workers and housewives. The routine data on which the analysis was based did not permit adjustment for likely confounding factors other than age and gender. The results regarding housewives may have been particularly vulnerable to unadjusted confounding from factors such as BMI, parity and past working history, which could have contributed to an overestimate of risk. Thus, we think that our findings regarding housewives should primarily be considered a stimulus for further study. Clearly, investigation of possible aetiological relationships between manual work and body weight^11^ were outside the scope of the study.

Possible discrepancies in blue-/white-collar/housewife classification between cases and the general population must be considered. After exclusion of military personnel and subjects with unknown active occupational status, the occupational groupings used for the census data and the hospital discharge records were readily classifiable into blue-/mixed collar and white-collar status. However, it is likely that both the hospital discharge records and census data contain some incorrectly coded information (plausibly leading to some non-differential misclassification). Of note, we do not think that differential classification of housewives should be a major concern since both the hospital discharge records and the census data provide specific categories for full-time housewives.

**Main messages**

- Manual work-related risk factors for surgical treatment of idiopathic carpal tunnel syndrome (CTS) appear to be relevant throughout working life for both men and women.
- As an occupational category, full-time housewives seem to have a raised risk of surgically treated CTS, possibly related to domestic work.

**Policy implications**

In aetiological studies of carpal tunnel syndrome, attention should be paid to domestic chores as a potentially relevant risk factor.

Although we were able to exclude repeated admissions with a principal diagnosis of CTS during the study period, we had no reliable way of identifying previous admissions before the study period (an issue likely to mainly effect the early part of the 4-year study period): thus the rates recorded are likely to be a slight overestimate. Of note, a sensitivity analysis based on the last 2 years of the study period did not show signs of distortion due to the inevitable inclusion of some prevalent cases. As regards the external validity of the findings, it is noteworthy that the overall rates of surgical treatment were broadly in line with those reported in other population-based surveys.^10^ ^24^ ^27^ However, the relative frequencies of surgery in the three occupational categories may have been influenced by the composition of the Tuscan workforce (including the distribution of blue-collar jobs) and possibly by the particular domestic culture of Italian housewives which includes elaborate cooking procedures and much house cleaning.

## CONCLUSIONS

In conclusion, blue-collar workers appear to have much higher rates of surgically treated idiopathic CTS than white-collar workers, irrespective of gender and age group. It is not easy to attribute such discrepancies exclusively to confounding factors such as differential body weight. Thus, the present work seems to underline the relevance of occupational risk factors for surgically treated CTS. Moreover, the remarkably similar patterns recorded for full-time housewives suggest that the role of domestic chores deserves more investigation as a possible risk factor for CTS (not only in full-time housewives). In conjunction with analysis of the possible risks associated with domestic duties, study of the biomechanical profiles of manual domestic chores could also be informative.
